# Investigation of Associated Gas-Assisted Surfactant-Polymer Flooding for Enhanced Oil Recovery in Heavy Oil Reservoirs

**DOI:** 10.3390/polym17233168

**Published:** 2025-11-28

**Authors:** Wei Wang, Xi Yan, Dandan Cui, Tao Song, Jianqiang Zi, Tenglong Sun, Yiqiang Li, Zheyu Liu

**Affiliations:** 1 Oil Production Technology Research Institute, Dagang Oilfield Company, PetroChina, Tianjin 300450, China; 2 State Key Laboratory of Petroleum Resources and Prospecting, China University of Petroleum (Beijing), Beijing 102249, Chinazheyu.liu@cup.edu.cn (Z.L.); 3 College of Petroleum Engineering, China University of Petroleum (Beijing), Beijing 102249, China

**Keywords:** heavy oil, high water-cut reservoir, SP flooding, associated gas-assisted flooding, EOR

## Abstract

Prolonged water injection in conventional heavy oil reservoirs typically leads to high water cut and a substantial reduction in recovery rate. This study explores the synergistic effects of a composite flooding process, where associated gas-assisted surfactant-polymer (SP) flooding is enabled by prior gel conformance control, to enhance oil recovery in these reservoirs. Through high-temperature, high-pressure microscopic visualization experiments and heterogeneous core flooding tests, the oil displacement mechanisms and enhanced recovery effects of this composite system were systematically investigated. The results show that SP flooding, through viscosity enhancement and reduction in interfacial tension, achieves the highest microscopic oil displacement efficiency, with an oil recovery of 81% and a significant reduction in clustered residual oil to just 9%. Associated gas flooding improves oil mobility by reducing viscosity and promoting expansion through gas dissolution, resulting in a recovery efficiency of 62%, which outperforms traditional viscosity reducers (58%). Heterogeneous core flooding experiments demonstrate that a composite strategy involving gel plugging, associated gas assistance, and SP flooding increases recovery by 24% compared to water flooding. The system also exhibits excellent flow control and maintains a low water cut, confirming the promising potential of this gel-conformance-controlled, associated gas-assisted SP flooding strategy as an effective method for enhancing recovery in high-water-cut heavy oil reservoirs.

## 1. Introduction

High-water-content conventional heavy oil reservoirs, a crucial oil and gas resource, face significant development challenges in various oil fields worldwide, including Canada, Venezuela, China, and parts of the Middle East [1,2,3,4,5]. These reservoirs, after prolonged water injection, often suffer from inefficient water cycling and low oil recovery rates, which drastically reduce economic benefits [6,7,8,9,10]. The core issue stems from the significant viscosity ratio between heavy oil and displacement water, leading to severe fingering and bypass flow [11,12,13]. As a result, a substantial amount of oil remains trapped in unswept middle-to-low permeability layers and microscopic pore throats. Traditional water flooding and standalone enhanced oil recovery (EOR) techniques have proven largely ineffective in addressing this issue [14].

In response to these challenges, the industry has explored several technical approaches, such as polymer/surfactant binary flooding [12,15,16,17], gas flooding [18,19,20], and chemical viscosity reduction [21,22,23]. Surfactant-polymer binary flooding shows considerable potential due to its ability to synergistically control fluid viscosity and reduce interfacial tension [24,25,26,27]. Associated gas, as an easily accessible and cost-effective displacement medium, has garnered significant attention for its ability to reduce viscosity through dissolution and enhance oil mobility [28,29]. Moreover, the use of associated gas for flooding offers an added environmental benefit by potentially reducing greenhouse gas emissions, as it utilizes gas that would otherwise be flared or vented [7,8,30]. Additionally, it can help lower oilfield development costs by using an existing resource, thus making the entire process more economically sustainable [31,32].

However, despite these advantages, these individual technologies have inherent limitations [33,34,35]. For instance, SP flooding often suffers from ineffective cycling in high-permeability channels, preventing it from reaching its full potential [36,37,38,39]. In terms of operational efficiency, mechanical shearing during injection and irreversible retention within the reservoir through adsorption and entrapment can markedly reduce polymer mobility control and increase operational costs [40,41,42,43]. Gas flooding, while promising, is constrained by the low viscosity of gas, which causes channeling and reduces sweep efficiency [44,45,46]. In addition to these methods, chemical viscosity reducers and gels are also pivotal in heavy oil recovery. Chemical viscosity reducers, often surfactant-based, function by adsorbing at the oil-water interface to emulsify the heavy crude into low-viscosity oil-in-water emulsions, thereby significantly reducing the oil’s flow resistance and enhancing displacement efficiency within swept areas [47,48]. Conversely, gels are primarily employed for conformance control. Typically formed by crosslinking polymers (e.g., polyacrylamide) with organic or inorganic crosslinkers, gels are designed to plug high-permeability “thief zones.” This diversion of subsequent displacing fluids into previously unswept, oil-bearing zones of lower permeability dramatically improves volumetric sweep efficiency [49,50]. Nonetheless, the effectiveness of viscosity reducers can be hampered by adsorption and limited penetration depth, while gel treatments require precise placement to avoid damaging productive zones. The fundamental mechanisms and constraints of polymer flooding, including adsorption and retention, are comprehensively detailed in foundational texts [51,52] and discussed in reviews [53]. Therefore, the development of a composite technical system that can synergistically expand swept volume, improve oil displacement efficiency, and offer both economic and environmental benefits has become a central research focus in the field of heavy oil development.

Indeed, several composite techniques have been explored to this end. Beyond surfactant-polymer (SP) flooding [54], other well-known methods include alkali-surfactant-polymer (ASP) flooding [55], which leverages alkali to generate in situ surfactants and reduce surfactant adsorption; water-alternating-gas (WAG) and its variants like foam-assisted WAG (FAWAG) [56], which aim to improve gas sweep efficiency; and simultaneous injection of miscible gas and polymers (SIMGAP) [57], designed to combine mobility control with miscible displacement. While these techniques show promise, challenges remain, such as alkali-induced scaling in ASP, the complexity and cost of foam generation in FAWAG, and ensuring effective synergy in simultaneous injections. The “associated gas-assisted SP flooding” system investigated in this study proposes a novel combination, specifically utilizing readily available associated gas as a synergistic agent with the SP system. In contrast to strategies that leverage viscous crossflow between fingers, our approach prioritizes frontal stability through a favorable mobility ratio (M~1) to address the specific challenges of heterogeneous, high water-cut reservoirs [58]. It seeks to integrate the robust mobility control and displacement efficiency of SP flooding with the deep-reaching viscosity reduction and swelling effects of gas, while potentially mitigating gas channeling through strategic slug placement and the presence of the polymer. This approach aims to offer a practical and efficient composite strategy, particularly for high-water-cut heavy oil reservoirs where associated gas is an accessible resource.

This study investigates a typical high-water-cut heavy oil reservoir (with a dead oil viscosity of <1000 mPa·s at the reservoir temperature of 78 °C) in the Dagang Oilfield, China, with the aim of clarifying the oil recovery mechanisms and synergistic effects of a composite system where associated gas-assisted SP flooding is enabled by an initial gel conformance control stage. By employing high-temperature, high-pressure microscopic visualization and heterogeneous core flooding experiments, this study systematically examines the microscopic displacement mechanisms of various media and validates the technological superiority of the composite system [16,59,60]. The findings of this research are expected to provide a potential solution to the development challenges faced by similar reservoirs in China and offer valuable insights for heavy oil reservoirs worldwide, by demonstrating the potential of a composite technique to address both technical challenges and environmental concerns.

## 2. Experimental Equipment and Methods

### 2.1. Experimental Materials

**Figure 1:**
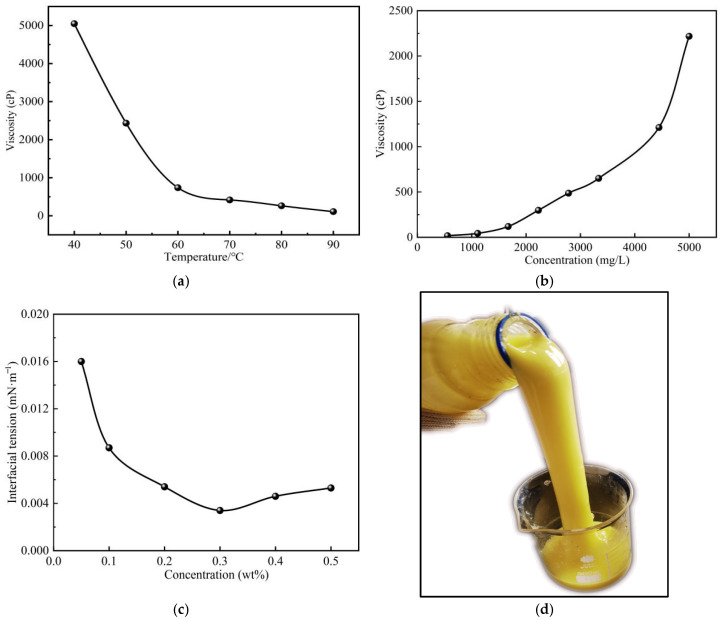
Key properties of the chemical flooding system. (**a**) Viscosity-temperature curve of crude oil. (**b**) Polymer viscosity-concentration curve (T = 78 °C). (**c**) The influence of concentration of surfactant to oil/water interfacial tension (T = 78 °C). (**d**) Gel morphology after 2 days of aging.

The crude oil employed in the experiments was conventional heavy oil obtained from the Dagang Oilfield. Under reservoir conditions of 78 °C, the oil exhibited a viscosity of 277 mPa·s and a density of 0.827 g/cm^3^, as illustrated in Figure 1a. The experimental water was entirely synthesized in the laboratory to replicate the ionic composition and salinity of the formation water in the study area, with a total salinity of 26,944 mg/L. The detailed ionic composition is provided in Table 1. As methane constitutes approximately 80% of the associated gas in the Dagang Oilfield, pure methane was used in this study. This approach avoids compositional variability, thus ensuring stable and repeatable experimental conditions, and establishes a conservative baseline for assessing gas-assisted recovery, since heavier hydrocarbons would likely enhance the performance. The polymer-surfactant binary system was formulated with a 2000 mg/L solution of an associative polyacrylamide (commercially known as BHAP-90) and a 0.2 wt% compound surfactant system comprising anionic and nonionic components (commercially known as 109-type). The viscosity reducer (VR) used was a nanoparticle-based viscosity-breaking agent (commercially known as 601-type additive). This AP was chosen for its exceptional salt tolerance, which is critical for maintaining solution viscosity and effective mobility control in the high-salinity formation brine, where conventional polymers like HPAM would suffer severe performance degradation. The gel used in this study was formulated by aging a solution of associative polyacrylamide and an oligomeric polyphenol crosslinker for two days at the reservoir temperature of 78 °C. This process resulted in a robust, high-strength network (15,000–20,000 mPa·s) through a combination of covalent crosslinking and physical associative interactions. The primary objective of deploying this gel was to selectively shut off the high-permeability zones. The gelant was designed with a viscosity lower than the reservoir oil to ensure its preferential entry into these water-flushed thief zones during injection, while the delayed gelation provided adequate time for deep placement before solidification.

### 2.2. Microfluidic Experiments

To elucidate the mechanisms of heavy-oil mobilization within reservoir pore structures under water flooding, viscosity-reducing (VR) flooding, SP flooding, and associated gas-assisted flooding, a series of separate, independent micro-scale displacement experiments were performed. Each experiment used a identical microfluidic chip to displace the oil with a single, specific displacement medium. This parallel design allows for a direct comparison of the intrinsic displacement efficiency of each medium without interference from previous flooding stages.

**Figure 2:**
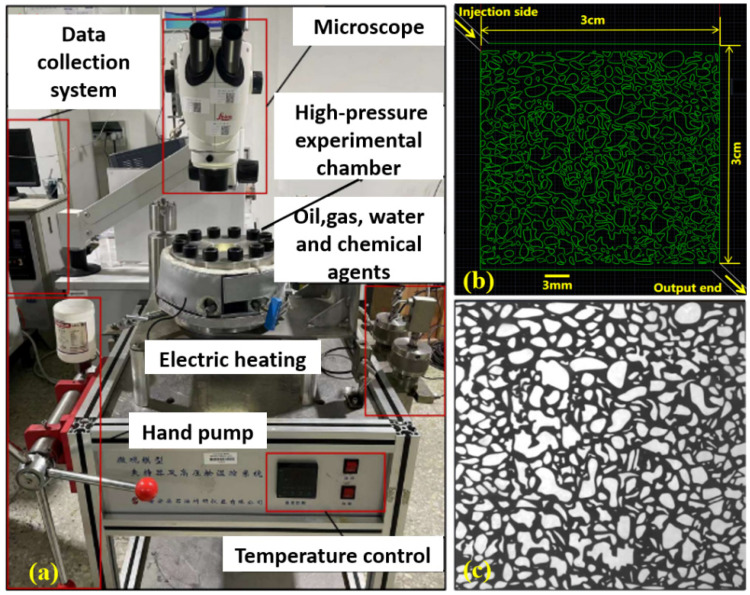
High-temperature and high-pressure microscopic visualization experiment. (**a**) Experimental setup assembly; (**b**) Microfluidic chip design diagram; (**c**) Observed chip in the viewing window.

The high-temperature, high-pressure microfluidic system (Huabao Co., Yangzhou, China; rated to 180 °C and 50 MPa) was used. Figure 2a illustrates the experimental setup, and Figure 2b,c depict the design layout and the oil-saturated microfluidic chip, respectively. The chip (Wenhao Microfluidic Technology Co., Ltd., Suzhou, China) was fabricated by glass etching to replicate the real pore-throat structure observed in reservoir thin sections, with dimensions of 3 cm × 3 cm × 20 μm (length × width × depth). The experimental procedure for each independent test was as follows:

(1) The microfluidic chip was installed in a high-temperature, high-pressure chamber, and the bolts were securely tightened. The displacement fluid was injected into a piston container, completing the experimental flow circuit.
(2) The model was vacuumed for 0.5 h with a vacuum pump, after which the temperature was raised to 78 °C and the chip was saturated with the experimental oil. The pressure was then increased to 20 MPa to simulate reservoir conditions, and the system was aged for 2 h.
(3) A single displacement medium (e.g., water, VR, SP, or associated gas) was injected into the model at a constant flow rate of 2 μL/min to initiate the displacement test. The experiment ended once the internal fluid distribution stabilized. Displacement dynamics were recorded in real time with a high-resolution microscope (Leica Microsystems, Wetzlar, Germany). The oil phase was distinguished from the aqueous phase and the solid matrix based on its high native optical contrast (appearing dark) under transmitted light, without the use of fluorescent dyes. The recorded images were then post-processed and analyzed using Photoshop 2024 and MATLAB 2022 to determine the oil saturation by applying a pixel intensity threshold for phase identification.
(4) After each run, the apparatus was thoroughly cleaned to remove residues and prevent cross-contamination. The same procedure was repeated for subsequent flooding schemes, including VR flooding, SP flooding, and associated gas flooding.

### 2.3. Core Flooding Experiments

**Figure 3:**
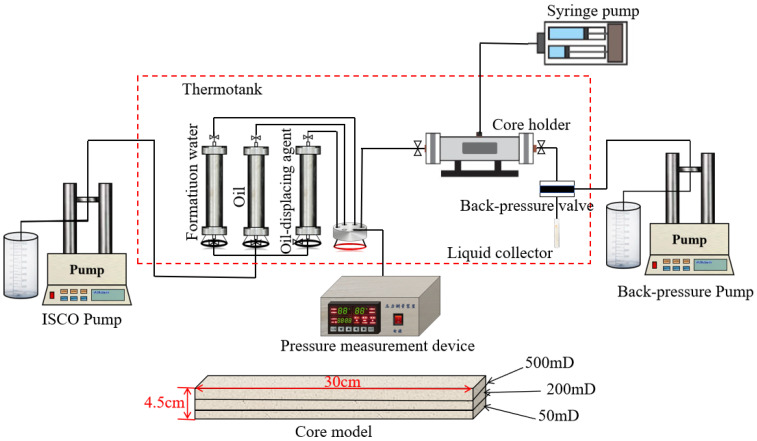
Experimental flowchart and the used core model.

To further elucidate the mechanisms and oil recovery efficiency of associated gas-assisted polymer–surfactant flooding, comparative displacement experiments were conducted using heterogeneous cores. The experimental design and core parameters are summarized in Table 2. The heterogeneous cores were self-fabricated artificial sandstone samples. They were prepared in-house using a large core pressing machine by sequentially packing and cementing quartz sand (mineralogy > 99% quartz) of specific grain sizes. The three distinct permeability layers—500 mD (upper), 200 mD (middle), and 50 mD (lower)—were achieved by controlling the sand grain size distribution and the applied compaction pressure. The cores were fabricated as rectangular prisms measuring 30 cm × 4.5 cm × 4.5 cm (length × width × height) with an average porosity of 26% ± 1%. The interfaces between layers are hydraulically connected, simulating a heterogeneous reservoir environment that allows for crossflow. Figure 3 shows a schematic diagram of the core flooding experimental procedure. The main equipment included a constant-temperature oven, a core holder, ISCO pumps, intermediate containers for formation water, crude oil, surfactant polymer solution, gel, and associated gas, a confining pressure pump, a pressure monitoring system, a back-pressure valve, and measurement instruments. The core flooding apparatus was manufactured by Huabao Co., Ltd., Yangzhou, China. The detailed experimental procedure is outlined below.

(1) Determine the gas permeability of the cores. The cores were vacuumed, saturated with water for more than 24 h, and the pore volume was calculated.
(2) Saturate the cores with oil at a low flow rate, determine the initial oil saturation, and age the cores at 78 °C in an oven for 24 h.
(3) Conduct the core flooding experiment: water flooding was carried out until a consistent volume of 1.0 pore volume (PV) was injected, at which point the water cut at the outlet had reached 95% ± 1%. This established a uniform baseline, after which the injection of various displacement media commenced.
(4) Record the produced fluid volumes and pressure data throughout the experiment, continuing the displacement until no oil production was observed.
(5) Replace the cores and conduct additional experimental schemes, repeating the above procedure for each test.

**Table 2:** Scheme and parameters of heterogeneous core flooding experiments.

Case	Permeability/mD	Oil Saturation	Experimental Scheme
1	500/200/50	0.67	1 PV water + 1 PV VR + post-water
2	500/200/50	0.66	1 PV water + 1 PV Gas + post-water
3	500/200/50	0.68	1 PV water + 1 PV SP + post-water
4	500/200/50	0.70	1 PV water + 0.1 PV Gel + 0.15 PV Gas + 0.3 PV SP + 0.15 PV Gas + 0.3 PV SP + post-water
5	500/200/50	0.67	1 PV water + 0.1 PV Gel + 0.15 PV VR + 0.3 PV SP + 0.15 PV VR + 0.3 PV SP + post-water

The design of the composite slugs in Cases 4 and 5 follows a strategic sequence to maximize synergistic effects and improve sweep efficiency. The process begins with an initial gel slug (0.1 PV) for deep conformance control, aiming to plug high-permeability thief zones and divert subsequent fluids into previously unswept, oil-bearing zones. This is followed by alternating injections of gas/VR slugs (0.15 PV) and SP slugs (0.3 PV) over two cycles. The gas/VR slugs function as mobility improvers, penetrating the newly accessed zones to reduce oil viscosity and create a mobilized oil bank. The subsequent SP slugs then act as efficient displacers and oil-washing agents, leveraging their high viscosity and ultralow interfacial tension to push the mobilized oil and scrub residual oil from pore walls. This split-slug, multi-cycle approach promotes a more stable displacement front and enhances both macroscopic sweep and microscopic displacement efficiency more effectively than a single, large slug injection.

## 3. Result and Discussion

### 3.1. Comparison of Microscopic Oil Displacement Effects

**Figure 4:**
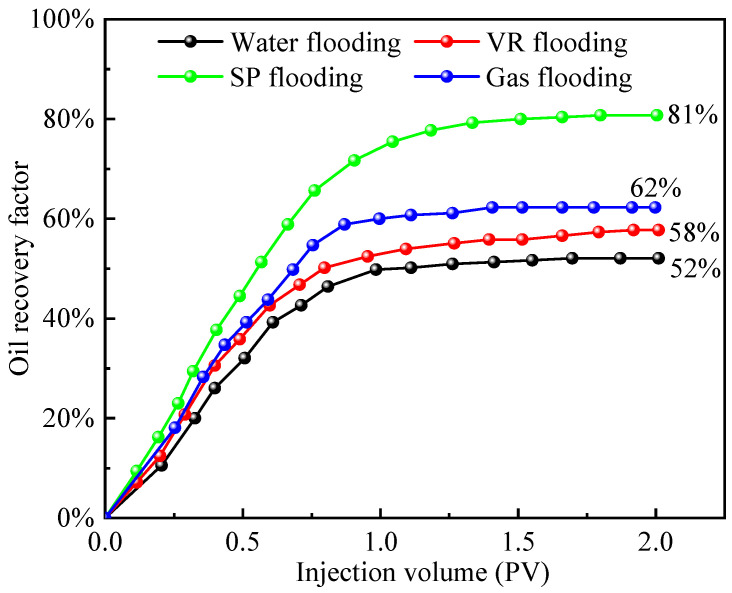
Variation in oil recovery with injected pore volume for different displacement media.

Quantitative analysis of residual oil distribution following the micro-scale displacement experiments was conducted using image recognition software. The pixel area of residual oil within the field of view was identified and expressed as a fraction of the model’s total pore-throat area, serving as an indicator of oil recovery efficiency. Figure 4 illustrates the variation in oil recovery with respect to injected pore volume (PV) for four different displacement media. Oil recovery increased rapidly during the initial stages as the injection volume increased. As the injected volume approached 1 PV, the rate of increase slowed markedly, indicating that the displacement process had gradually entered the residual oil mobilization stage. Comparison of final oil recovery across the different systems indicates that SP flooding exhibited the most effective mobilization of oil within the pore network, achieving 81% recovery. Associated gas flooding resulted in 62% recovery, VR flooding achieved 58%, and water flooding was the least effective, yielding only 52% recovery. The SP flooding recovery of 81% approaches the practical limit for this heterogeneous pore system, with residual oil trapped in inaccessible pores. The 52% water flooding recovery, higher than the macroscopic viscosity ratio would suggest, is attributed to pressure-driven crossflow enabled by the 2D pore network.

**Figure 5:**
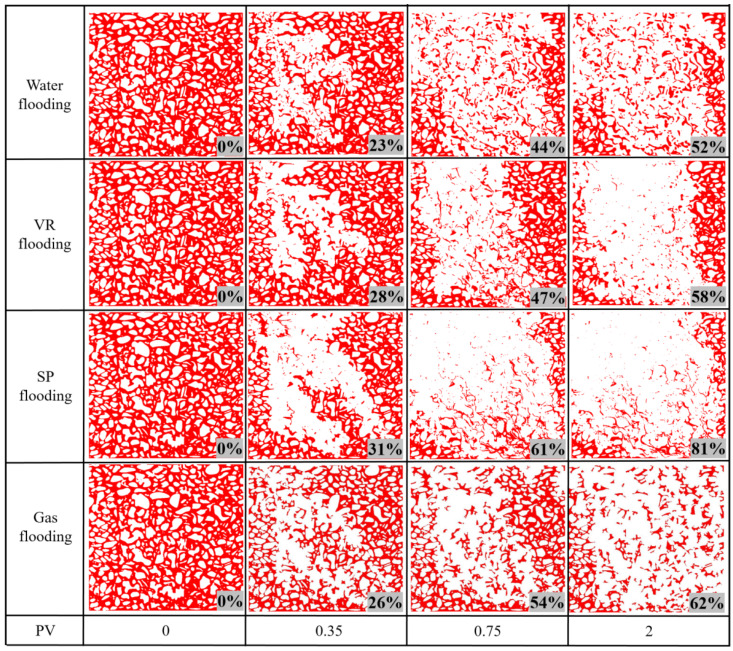
Change in the oil (in red) configuration in the analyzed part of the microchip as a function of pore volume (PV) injected.

Figure 5 illustrates the morphological distribution of residual oil with respect to injected pore volume for four different displacement media. Red areas represent residual oil, and white areas correspond to the rock matrix and the swept regions. The extent of oil recovery is primarily governed by the sweep efficiency and displacement efficiency of the injected fluids. Comparison of sweep efficiencies reveals significant differences among the systems, ranked as SP flooding, associated gas flooding, water flooding, and VR flooding; these differences are mainly controlled by the combined effects of mobility control and interfacial behavior. SP flooding optimizes the mobility ratio by markedly increasing the viscosity of the displacing phase via the polymer, while the surfactant facilitates ultralow interfacial tension and emulsion transport. Their synergistic effect ensures the most extensive macroscopic and microscopic sweep. Although associated gas flooding improves oil mobility via viscosity reduction and oil swelling, its low intrinsic viscosity makes it susceptible to gas channeling and gravity override, thus limiting sweep efficiency. Water flooding shows low sweep efficiency, owing to a highly unfavorable mobility ratio with heavy oil, resulting in pronounced viscous fingering.

Notably, VR flooding achieves lower sweep efficiency than associated gas flooding, primarily due to fundamental differences in mechanism and microscopic sweep behavior. Associated gas flooding relies on gas components dissolving into oil under high pressure, resulting in global viscosity reduction and volumetric expansion of heavy oil, thus systematically enhancing oil mobility. In contrast, VR flooding is highly localized and interfacially dependent. It primarily reduces viscosity via adsorption and emulsification at the oil-water interface, with a limited effective radius in the porous medium and rapid loss due to adsorption of its chemical components onto rock surfaces, thereby hindering deep sweep. Furthermore, some emulsifying viscosity reducers can form high-viscosity emulsions in micro-pores [61], inducing severe jamming effects, locally increasing flow resistance, diverting the displacing fluid, and exacerbating heterogeneous fingering, ultimately reducing macroscopic sweep. The overall displacement efficiency, which integrates both macroscopic sweep and microscopic displacement efficiency, decreases in the order of SP flooding, associated gas flooding, VR flooding, and water flooding (Figure 5). This ranking is determined by the competing effects of mobility control and interfacial behavior on the two efficiency components.

### 3.2. Characteristics of Microscopic Residual Oil

**Figure 6:**
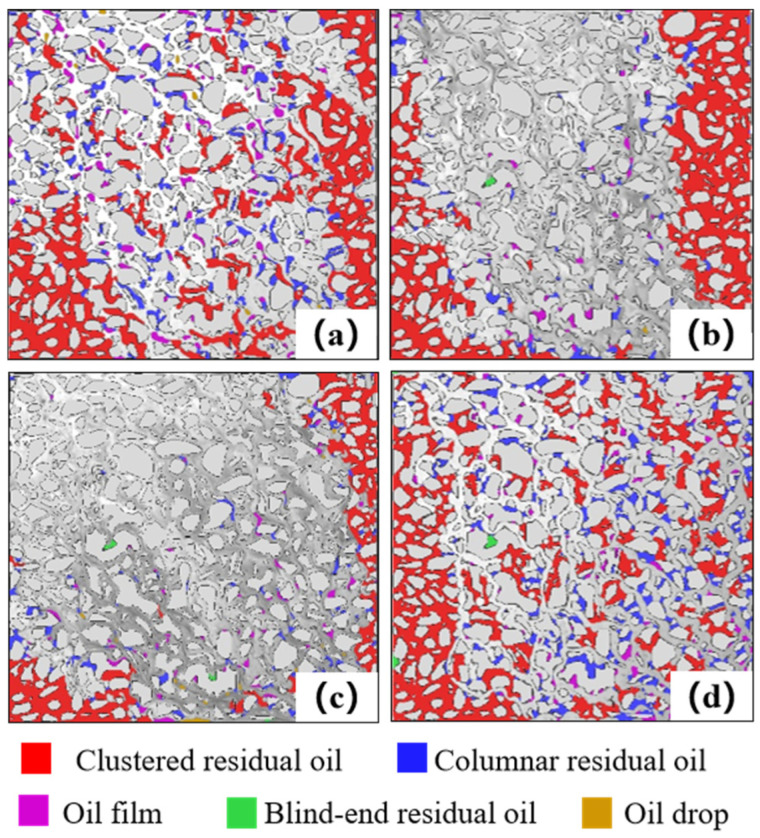
Images of microscopic residual oil after displacement with four different fluids. (**a**) Water flooding; (**b**) VR flooding; (**c**) SP flooding; (**d**) Gas flooding.

**Figure 7:**
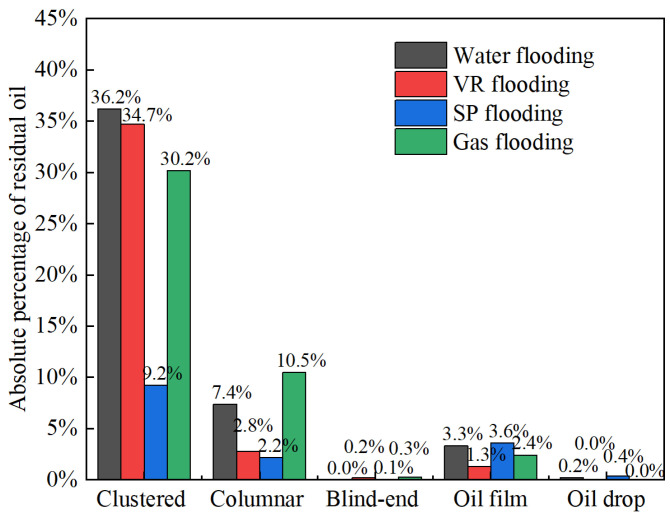
Absolute proportion of residual oil types after displacement by four different flooding fluids.

Residual oil within the micro-pore-throat network was identified and classified using a process that combined MATLAB-based image processing with subsequent morphological analysis and manual verification. The oil phase was first segmented from the background by applying a pixel intensity threshold. The classified oil blobs were then analyzed based on morphological characteristics (e.g., shape, size) and their spatial relationship with the pore structure to assign them to predefined types (clustered, columnar, etc.), following established visual criteria. While the high image contrast ensured robust identification of dominant morphologies, we acknowledge that classifying complex or transitional shapes involves a degree of subjectivity. To ensure consistency, the final classification was performed by a single experienced researcher, and its occurrence state was determined according to its morphology and contact with pores and grains. Residual oil was categorized into five types: clustered residual oil, columnar residual oil, oil films, Blind-end residual oil, and oil drop. Clustered residual oil refers to isolated oil globules surrounded by water in multiple pores, whereas columnar residual oil refers to oil columns in relatively large pore-throats, trapped between two or more water segments. Figure 6 shows the residual oil identification results after displacement by four different flooding media. To quantitatively compare oil mobilization by each medium, the absolute proportion of each residual oil type relative to the total saturated oil was calculated, as shown in Figure 7. The proportion of clustered residual oil increases in the following order: SP flooding (9%), associated gas flooding (30%), VR flooding (35%), and water flooding (36%). A smaller proportion of clustered residual oil indicates higher sweep efficiency, suggesting that the SP system achieves the most extensive sweep. Columnar residual oil reflects the ability of the displacement medium to reduce oil-water interfacial tension. The SP system performs comparably to the VR, whereas associated gas flooding shows the weakest reduction, with columnar residual oil accounting for 10%. The proportion of oil-film residual oil indicates the ability of the displacement system to detach oil adhered to pore surfaces. The SP and VR systems exhibit stronger detachment, followed by associated gas flooding, and finally water flooding.

### 3.3. Comparison of Displacement Characteristics in Heterogeneous Cores

**Figure 8:**
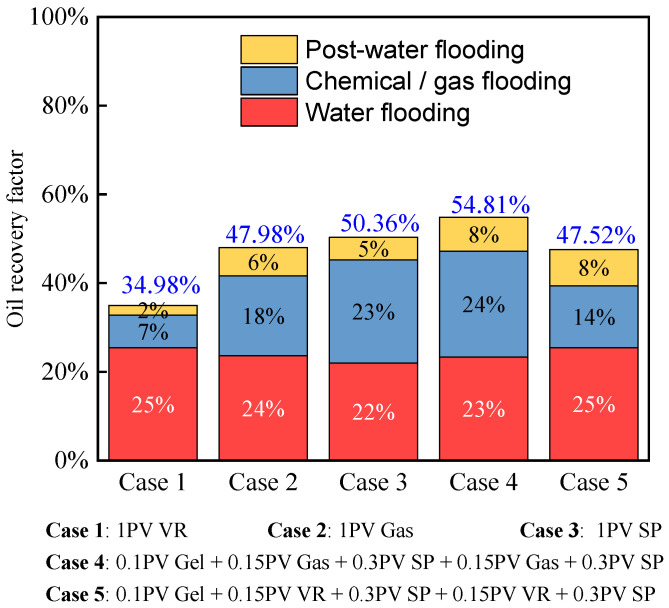
Comparison of oil recovery at various stages during oil displacement experiments in a heterogeneous long core.

Figure 8 presents a comparison of oil recovery from three-layer heterogeneous long-core experiments. In Scheme 1 and Scheme 2, associated gas flooding following water flooding achieved higher recovery than viscosity-reducing flooding following water flooding. Comparison of Scheme 3, Scheme 4 and Scheme 5 indicates that in Scheme 4, associated gas flooding applied during multi-stage slug injection achieved the highest recovery of 24%, and the subsequent water flooding still contributed significant incremental oil recovery. Although viscosity reducers were used in Scheme 5, oil recovery remained sub-optimal, even lower than that achieved by SP flooding, suggesting that in non-thermal heavy oil recovery from heterogeneous reservoirs, expanding the swept volume remains the primary challenge to be addressed.

**Figure 9:**
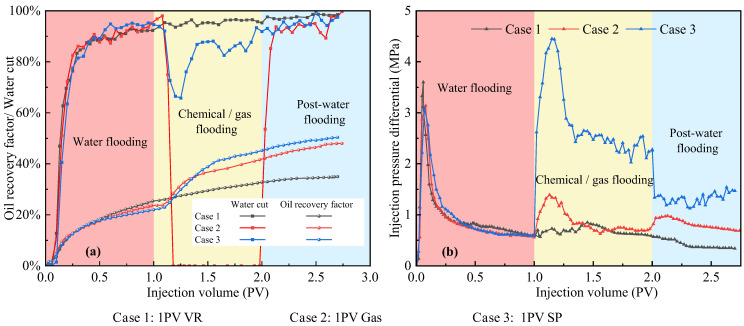
Comparison of core flooding results using a single displacement fluid. (**a**) Oil recovery and water cut as functions of injected pore volume; (**b**) Injection pressure difference as a function of injected pore volume.

Figure 9 presents the production characteristic curves for experiments where a single displacement fluid was injected after water flooding. Upon entering the chemical or gas flooding stage, marked differences in oil recovery were observed among the schemes. VR flooding primarily enhances flow by reducing oil viscosity; however, when injected after water flooding, it tends to channel through high-permeability paths, limiting contact with oil and diminishing its effectiveness. Associated gas flooding, despite encountering water-filled channels that reduce its relative permeability, can penetrate low-permeability zones bypassed by water due to its strong molecular diffusion, simultaneously lowering oil viscosity and generating dissolved gas drive, thereby improving oil mobility. Nevertheless, late-stage gas channeling still restricts overall recovery. SP flooding provides stable mobility control and efficient microscopic displacement through the synergistic effects of polymer-induced viscosity increase and surfactant-induced interfacial tension reduction, as evidenced by continuously decreasing water cut and steadily increasing oil recovery. Comparison of the injection pressure curves (Figure 9b) indicates that SP flooding exhibits the highest pressure drop, demonstrating its superior mobility control and microscopic displacement capability. Associated gas flooding exhibits a moderate pressure drop due to the combined effects of low gas viscosity and pore-throat jamming, whereas VR flooding displays the lowest pressure drop due to its limited capacity to increase the viscosity of the displacing phase and improve the mobility ratio.

**Figure 10:**
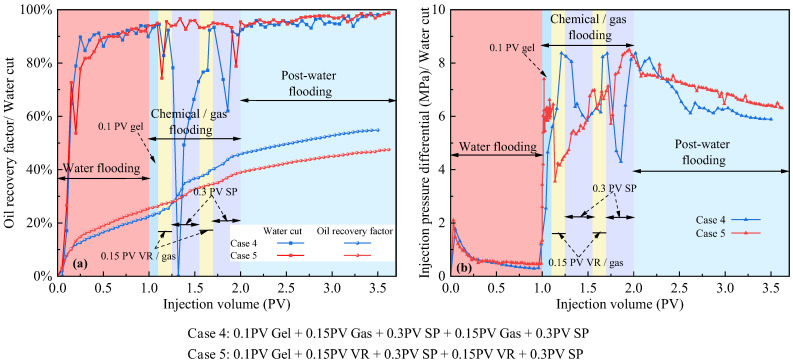
Comparison of core flooding results using multiple displacement fluid slugs. (**a**) Oil recovery and water cut as functions of injected pore volume; (**b**) Injection pressure difference as a function of injected pore volume.

Figure 10 presents the production characteristic curves for multi-medium composite flooding following water flooding. The design of the composite slugs in Cases 4 and 5 follows a strategic sequence to maximize synergistic effects. The initial gel plugging (0.1 PV) diverts subsequent fluids into unswept zones. The following alternating slugs of gas/VR and SP are designed to work in concert: the gas/VR slug penetrates into these newly opened zones, reducing oil viscosity and improving mobility to create an oil bank. The subsequent SP slug then effectively displaces this mobilized bank and scrubs residual oil due to its high viscosity and ultralow interfacial tension. This split-slug, multi-cycle approach enhances both macroscopic sweep and microscopic displacement efficiency more effectively than a single, large slug injection. The flow diversion was quantitatively evidenced by the system’s pressure and production response. As shown in Figure 10, gel injection caused a sharp pressure rise from 0.5 MPa to 8 MPa, confirming effective plugging. This was immediately followed by a significant increase in oil recovery, providing direct evidence that the diverted fluid mobilized additional oil from unswept zones. Subsequently, alternating injections of associated gas and the SP system were carried out. By leveraging the superior sweep diffusion and viscosity-reducing properties of associated gas, together with the mobility control and emulsion-assisted oil transport capabilities of the SP system, two rounds of injection maximized the mobilization of previously unswept oil. In high-water-cut, conventional heavy oil reservoirs, gel plugging followed by associated gas-assisted SP flooding provides substantial economic benefits and favorable recovery performance. The key lies in gel blocking dominant channels, with the synergistic action of associated gas and the SP system forming a more efficient three-dimensional composite displacement strategy, effectively suppressing gas channeling and viscous fingering.

It is also important to note that the strong incremental recovery observed during the post-EOR water injection stage is not a result of water alone, but rather the consequence of reservoir conditioning induced by the preceding gel, gas, and SP slugs. The gel preferentially plugs the high-permeability thief zones, while the subsequent gas/SP slugs alter the local mobility ratio, redistribute fluid paths, and mobilize oil in low-permeability regions that were previously bypassed during initial water flooding. This sequential modification of the flow field forces the later water injection to enter newly swept regions instead of fingering through dominant channels, thereby generating additional oil production. This mechanism explains why, in Figure 9 and Figure 10, post-water injection continues to exhibit significant oil increments even in a heterogeneous core system.

It should be noted that the high injection pressures observed in these laboratory experiments would need to be carefully managed in field applications to avoid formation damage or fracturing. Operational strategies such as controlling injection rates or employing pre-conditioning slugs would be essential to maintain the pressure within a safe window.

## 4. Conclusions

This study systematically investigates the mobilization mechanisms and oil-displacement efficiency of various oil-displacement systems for a heavy oil system. The research methodology integrates microscopic visualization displacement experiments, quantitative analysis of remaining oil images, and displacement experiments using heterogeneous core samples. The main findings of the study are summarized as follows:

(1) SP flooding exploits the synergistic effects of polymer-induced viscosity increase and surfactant-induced interfacial tension reduction, achieving optimal mobility control and oil-washing efficiency in our experiments, with a final oil recovery of 81%. Its microscopic sweep and oil-washing performance outperformed all other schemes tested in study, mechanistically highlighting its strong potential as a primary displacement medium.
(2) Quantitative analysis of microscopic residual oil shows that SP flooding mobilizes all types of residual oil most effectively, reducing clustered residual oil to 9%, substantially lower than in VR flooding (35%) and water flooding (36%). Associated gas flooding exhibits moderate potential for improving the sweep of clustered residual oil, whereas VR flooding, despite its oil-washing capability, tends to induce jamming effects in porous media, thereby limiting its sweep efficiency.
(3) In heterogeneous core experiments, directly switching to VR flooding after water flooding yielded minimal incremental recovery. While associated gas flooding led to a noticeable oil response, its overall efficiency was constrained by early gas breakthrough and poor sweep efficiency. Preceding gel plugging, however, increased the recovery increment of subsequent composite flooding by up to 24%, clearly demonstrating that “profile modification prior to displacement” is crucial for expanding the swept volume and enhancing recovery in heterogeneous reservoirs.
(4) The composite system, which integrates gel conformance control with associated gas-assisted SP flooding, achieves profile modification, displacement, and oil-washing through multi-medium synergy. In our core flooding experiments, it achieved the highest injection pressure differential, exhibited effective flow control, and maintained a low water cut with high recovery across multiple injection cycles, demonstrating its promising potential for field application.

## Figures and Tables

**Table 1:** Ionic composition of formation water.

Ion	K^+^/Na^+^	Mg^2+^	Ca^2+^	Total
Concentration mg/L	10,304	49	190	26,944
Ion	Cl^−^	SO_4_^2−^	HCO_3_^−^	
Concentration mg/L	16,183	96	122	

## Data Availability

The data presented in this study are available on request from the corresponding author due to institutional and confidentiality restrictions.
